# Just Love Me, Feed Me, Never Leave Me: Understanding Pet Food Anxiety, Feeding and Shopping Behavior of US Pet Owners in Covidian Times

**DOI:** 10.3390/ani11113101

**Published:** 2021-10-30

**Authors:** Meike Rombach, David L. Dean

**Affiliations:** 1 Department of Land Management and Systems, Lincoln University, Lincoln 7647, New Zealand; 2 Department of Agribusiness and Markets, Lincoln University, Lincoln 7647, New Zealand; david.dean@lincoln.ac.nz

**Keywords:** COVID-19, pet food anxiety, pet food insecurity, pet parenting, panic buying

## Abstract

**Simple Summary:**

Food insecurity and anxiety is an important topic in Covidian times. This study proposes a model that investigates the impact of pet owner’s perceptions of their pet, their engagement with their pet, sociodemographic factors and the frequency of incidences where pet owners could not provide sufficient food for their pet. The results are relevant to vets, managers and volunteers at animal shelters and pet food pantries, as behavioral changes in feeding and pet food buying resulting from pet food anxiety require awareness and accommodation in the everyday life of food insecure pet owners.

**Abstract:**

The study provides insights for pet food retailers, vets and managers and volunteers at animal shelters, pet food pantries and food banks into the behavioral changes in feeding and pet food buying resulting from pet food anxiety in Covidian times. This study proposes a model that investigates the impact of pet owner’s perceptions of their pet, their engagement with their pet, sociodemographic factors and the frequency of incidences where pet owners could not provide sufficient food for their pet. For this purpose, an online survey with a sample of 206 US residents was conducted. Partial least squares structural equation modelling shows that perceiving the pet as an animal or family/friend, as well as active engagement with the pet, heightens a sense of pet food anxiety. Similarly, past experiences where pet owners could not provide sufficient food for their pet impacts pet food anxiety, which leads to changes in pet food shopping and pet feeding behavior. Sociodemographic factors (biological sex, age, income and education) were not found to impact anxiety.

## 1. Introduction

In the US, approximately 85 million households own a pet, which is reportedly the highest proportion of households with pets in the world. The US pet population consists of cats, dogs, birds, ponies, rabbits, fish, reptiles and guinea pigs [1]. Pets have an important role in society [2] as they contribute positively to mental well-being and are an important contributor to the economy [3,4,5,6,7]. Pets are commonly viewed as family members or as close friends [8,9] and can perform duties as service and emotional support animals, and are often important companions for the elderly, for people with disabilities, people living in social housing, soldiers and veterans, students and people affected by autism, homelessness and unemployment [10,11,12,13,14].

Pets are commonly bought from breeders, animal shelters and pet stores [2,15,16,17]. Annual check-ups, health issues, microchipping and emergencies are common reasons for veterinary service utilization and they represent considerable expense [18]. Another significant expenditure for US pet owners is food [19,20]. Feline and canine pet owners typically have minimum expenses ranging from $254 to $287 per annum for basic pet food [21] and may pay an extra $72 to $81 for treats [1]. Other pet expenditures include toys and accessories; overall pet expenses have been growing steadily [22]. In 2020, US pet food and treat spending reached almost $42 billion [1].

The outbreak of COVID-19 has disrupted pet buying patterns and has changed the consumption patterns of pet owners for medical services and food items [18,23,24]. Arluke (2021) claims that COVID-19 has degraded the living conditions of 29 million pets living in low-income households, where food supply at times is insufficient due to limited household budgets and the difficulty of accessing stores [25]. Such households may seek assistance from animal welfare organizations and pet food pantries to assure regular feeding [26,27,28,29], but also to implement feeding behavior that aligns with human behavior displayed when suffering from food insecurity [25].

Following Rauktis et al. (2017) and Arluke (2021), behavioral coping mechanisms include buying low-quality food, bulk food, store brands or offers, or may even induce affected people to use coupons and shop for pet food in several different stores. More extreme behavior involves refraining from purchasing, delaying the payment of invoices, borrowing money, selling possessions and starving to ensure family members and pets have food [9,25].

Pet food shopping may cause pet owners increased stress and anxiety, due to the prevalence of panic buying by other customers, product shortages and limited access to animal welfare associations, food banks and food pantries due to COVID-19 restrictions. Therefore, the present study is dedicated to pet food insecurity and anxiety of pet owners when shopping for pet food, and builds on Rauktis et al. (2017) and Arluke (2021) [9,25]. The study aims to cover the interests of people involved in animal welfare and fill a literature gap by focusing on the key factors thought to influence US consumers’ feeding and shopping behavior, which has been impacted by COVID-19.

### 1.1. Factors Impacting Feeding and Buying Behavior

Previous studies have identified that the feeding and shopping behavior of US consumers is likely to be influenced by several factors: the sociodemographic backgrounds of consumers, their perception of their pet, their active engagement with their pet, the frequency of having sufficient pet food available and their experience of pet food anxiety. Given the relatively small body of literature on pet food insecurity and anxiety being attributed to COVID-19 in a US context, the literature review borrows from the wider research body dedicated to pet ownership in Europe, Australia and other countries and assumes similitude to the US concerning animal enthusiasm. It is expected that the key factors impacting buying and feeding behavior are similar, as these behaviors are essential to pet ownership per se. In addition, the COVID-19 pandemic led to disruption and adverse effects throughout the entire world.

### 1.2. Perception of Pets and Pet Engagement

An attitude is a psychological tendency that is expressed by evaluating a particular entity with some degree of favor or disfavor [30]. In the context of this study, it is the evaluation of whether the pet is perceived favorably as family/friend or somewhat unfavorably as an animal potentially providing services to human beings [31]. Given that perception commonly determines how engaged an owner is with a pet, an understanding of pet perception and pet engagement is needed.

### 1.3. Perception of Pets

Human–animal interactions and the perceptions and attitudes of US residents towards pets have been widely studied in recent years and are extremely diverse. The following section will discuss the perception of pets in a context of kinship/relationship, in terms of the service pets provide to their owners, in terms of their status as domesticated animals and the usefulness of pets to their owners.

In terms of kinship/relationship, Bir et al. (2016a) investigated the perceptions of US residents towards dogs in an animal welfare context. The study emphasized that US residents identified familial relationships with their pets. For example, pets were described as family members and viewed as foster children or grandchildren. It was further reported that dog owners had a duty to provide a good standard of care for their pets. Additionally, some survey participants had more extreme views and thought that dogs should have rights similar to human beings [31].

The study further discussed that US residents characterized their dogs depending on their values, beliefs and knowledge of animal welfare. These findings corroborate a recent study on cat welfare [31]. Bir et al. (2016b) indicate that perception and attitudes towards cats are likely to impact the form and quality of care that they receive [32]. These findings were confirmed by Ortez et al. (2018), who researched the public perception of animal welfare organizations in the US [33]. The authors stress that increased public concerns for animal welfare are a result of US residents identifying pets not only as animals but as family members [33].

With respect to service provision, another animal welfare study analyzed the perceptions of assistance and companion dogs among Australian citizens [34]. The results revealed that Australians were interested in both types of dogs, but more approving of the use of assistance dogs, and perceived them as happier. The perceived practical benefits of assistance dogs and the emotional benefits of companion dogs were acknowledged by Australian citizens. Differences in happiness ratings were attributed to publicly known cases of animal neglect of companion dogs, whereas there were no such incidences associated with assistance dogs [34]. These findings confirm an earlier study [35] that researched the public perceptions of service dogs, emotional support dogs and therapy dogs in the US. The results suggested confusion about definitions, rules, regulations and rights associated with each type of assistance dog. Service dogs were perceived as helping with a legitimate need and their access to public spaces was viewed favorably. Emotional support dogs were not well regarded. The study emphasizes the role of the media as contributing towards negative perceptions of emotional support dogs, as media coverage presents cases of abuse [35].

Izaguirre and Montiel (2021) focused on the status of pets as domesticated animals. They researched the perception of free-roaming cats and dogs on four university campuses in Mexico and the reported human–animal interactions [36]. Whereas most of the surveyed students have a positive perception of these animals and treat them similarly to their own pets by petting and feeding them, faculty members do not view them as pets and have reservations about touching and feeding them. Faculty members and administrators were the most concerned about dogs and cats projecting a bad image of the university. The study provides best-practice recommendations for managing free-roaming animals as there is a grey area about whether these animals are perceived as wild or domestic animals [36].

Further information on the usefulness of pets to their owner with respect to sport, guardiancy and companionship was provided by a European study. Ellingsen et al. (2010) researched dog owner empathy towards and the perception of their canine companions in Norway [37]. The results showed that pet owners viewed their dogs either as domesticated animals, friends or family. These differences in perception were based on the owner’s use of their dog, whether it was kept for companionship or hunting and household size as an indication of human social relations. Owners with a more positive perceptions of pets had higher empathy for their dogs [37].

Drawing from the previous studies, one’s knowledge of, attitudes towards and perception of a pet determines whether it is viewed as a domestic, wild or service animal, or a pet, a friend or a family member [31,36]. This view influences their engagement with, treatment and care of the animal.

### 1.4. Active Engagement with Pets

Haggerty-Davis et al. (2015) studied pet engagement from a pet care management perspective. The results of the study indicated that pet care is perceived as a chore-like duty in many households and differs according to family composition and situation. Pet engagement and care become a parental chore in families with preschoolers, a shared duty in households with schoolchildren and, in families with teenagers, the teenager is in charge of caring for the pet [38]. These findings confirm earlier studies, which indicate that a child is often involved in a pet purchase, but active pet engagement remains with the parents as the major pet caregiver [39].

Glansville et al. (2020) developed a pet care model with the pet owner being one of the most important contributors to pet welfare [40]. Engagement with the pet and care for the pet through playing, grooming and feeding are essential factors to animal welfare. The model builds on the pet owner’s values, beliefs and attitudes that underpin duty of care and contribute most strongly to an individual’s pet care competency. The model shall inform behavior change programmes that aim to understand owner motivation and improve their pet engagement behavior [40].

The tenets of the Glansville et al. (2020) model corroborated previous studies addressing non-compliant pet owners [40] who do not provide regular and good quality care for their pets. These owners engage in active pet cruelty [41,42,43] and passive cruelty, such as failing to provide physical activity, regular feed, grooming and veterinary visits [44].

Proper pet engagement and care is increasingly important in Covidian times [45,46], as extensive periods at home and insufficient economic resources may constrain the basic needs of a pet and influence human–animal interactions, with small children or adults being more strongly attached to pets [46]. The ability of the owner to provide sufficient care for and be appropriately engaged with the pet may be influenced by the sociodemographic profile of the pet owner.

### 1.5. Sociodemographic Backgrounds of Pet Food Consumers

Numerous studies on pet ownership have examined the sociodemographic backgrounds of pet owners. However, literature addressing links between specific concepts, such as pet food insecurity or anxiety with sociodemographic characteristics is limited. Furthermore, the conclusions drawn regarding pet ownership and sociodemographic information is inconclusive. In earlier studies, Gillum and Obisesan (2010) emphasize that pet ownership is associated with age, marital status, education, religion and mobility limitation [47]. More recent studies show that pet ownership and preferences for pet supplies and services including food are rather a matter of experience, attitudes and lifestyle [17,18,21]. Studies dedicated to pet food quality, pet food industries and pet food preservation and storage show that women are more likely to decide which pet food to buy [48,49,50]. Pets are present in 85% of all US households and ownership occurs throughout all social classes, across all ages, levels of income and education [1,17,21]. It is likely that people with low incomes, those who have migrated or have a refugee background or little education are those suffering from food insecurity and are more negatively affected by COVID-19 [23,25]. These people are coming from communities in need and under normal circumstances already rely on food assistance to get by [9,25]. Therefore, it can be expected that such individuals are experiencing increased stress and anxiety when buying pet food as they are exposed to additional pressure from product shortages and panic buying behavior by other customers [51,52].

### 1.6. Behavioral Adjustments to COVID-19

In addition to the adjustments required when buying pet food due to the presence of COVID-19, changes in feeding can be expected. Both types of behavioral changes and their linkage to anxiety are essential to understand when studying buying and feeding behavior in Covidian times.

### 1.7. Anxiety and Buying Behavior

Specific studies reporting consumer’s pet food preferences and buying behavior in 2020–2021 during the COVID-19 pandemic are rather limited [23,25]. However, numerous studies reported general changes in consumer behavior as a result of the disruptions of COVID-19 and restrictions on social life, including shopping [53,54,55,56,57]. These changes included several forms of unusual behavior leading to empty shelves and product shortages [51,52]. Water, toilet paper, hand sanitizer, pet food and other various staple products were scarce as they emerged as the preferred items for consumers who reacted to the stress and uncertainty with panic and bulk buying [58]. These reactions are not uncommon, as shown in previous studies on consumer behavior after extreme events, such as earthquakes and natural disasters [59].

The availability of product substitutes helped consumers to better cope with anxiety, [60,61] but seeing other customers bulk buying led to more competitive shopping behavior [62]. Bulk and panic buying are expressions of hoarding behavior, which is motivated by fear of unpreparedness and risk aversion stemming from evolutionary human instinct [59]. Given that consumers are used to the constant availability of products, including pet food, scarcity of these products and resulting competitive buying is viewed as a form of territorial behavior [59]. As pets are considered as family members, and that pet food is often bought when buying groceries, it is likely that these behavioral changes also affected pet food buyers.

A further behavior change was an increased preference for other forms of shopping, notably home delivery, click and collect options [57] and regular subscription services, and there were changes in views and preferences for branded products [59]. Physically distant shopping was encouraged by governments to minimize infection chains [55,63]. Pet supplies, such as accessories, toys and food were widely available online, and veterinary services tended more towards online consultation [18]. Online retail may provide pet food buyers more certainty as to whether or not pet food is available, which may prevent stress and anxiety caused by shopping experiences and consumer behavior in Covidian times.

The perception and purchase of some branded items changed as well [64,65]. Consumers tend to actively switch brands if the brands do not respond appropriately in a crisis. Brand and marketing communications that are too humorous or light hearted are considered inappropriate [59]. During the coronavirus pandemic, there was a focus on gratitude towards essential workers and compassion towards consumers while they were forced to stay at home [59]. Other reasons for brand switching were product scarcity or changes due to income constraints as a result of redundancy and unemployment [53]. Pet owners may have actively switched brands during the COVID-19 pandemic for affordability reasons or because they felt that the brand promise was betrayed. Inappropriate marketing communication and brand message may result in stress, irritation or increased frustration and lead to competitive or panic buying behavior.

### 1.8. Frequency of Food Insecurity and Changes in Feeding Strategies

While specific time frequencies for when US pet owners experience problems providing pet food for their feline and canine companions remain unclear, indications can be provided by studies addressing pet ownership and food insecurity. Arlurke (2021) outlines circumstances and times where pet owners were unable to provide sufficient food for their pets [25]. Situations include unexpected life events and related expenses and “end of the month” scenarios where household budgeting and food management failed. In these situations, pet owners employed coping mechanisms, such as stretching pet food with human food, providing less feed at each serving to extend the feeding period or allowing their pet to go hungry for a day or two. Pet owners seem to be reluctant to provide inexpensive low-quality food and would rather sacrifice the quantity of their food than expose their pets to food insecurity. Other situations where pet owners had to employ coping mechanisms, such as borrowing money, not paying bills or sending their pet on the streets to find food, were when pet owners could not access a store because they could not pay for transportation or could not afford to pay prices in a nearby store. Having insufficient pet food available until the next shopping opportunity or not having access to food pantries or food banks is a considerable worry for many pet owners on low incomes [25]. Feeding problems and coping mechanisms increased because COVID-19 brought increased hardship to US communities, particularly to people with low income or food insecurity problems, due to product shortages and restricted access to shops, food banks, pantries and animal welfare organizations [23].

### 1.9. Conceptual Framework

**Figure 1:**
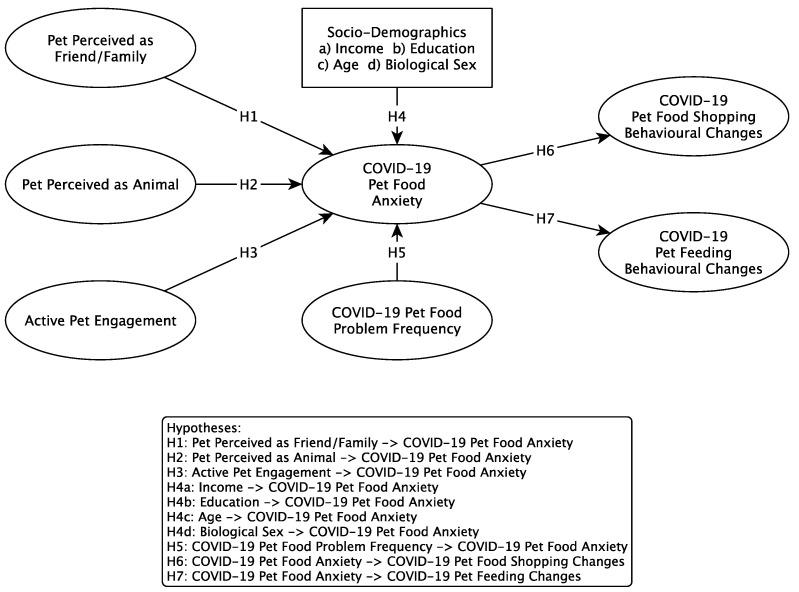
Conceptual Framework.

A conceptual framework building on the literature is proposed. It is suggested that behavioral changes in feeding and buying behavior are a result of pet food anxiety, which is influenced by factors such as the owner’s perception of their pet, their active engagement with their pet, their sociodemographic background and the frequency of incidences where they have been unable to provide sufficient food for their pet (see Figure 1). While the proposed conceptual framework attempts to incorporate all of the relevant research findings into a comprehensive set of relationships, the COVID-19 specific constructs and relationships do not have the same depth of literature support and are somewhat exploratory in nature. The following hypotheses are proposed:

**Hypothesis 1** **(H1).** *Pet owners who perceive their pet as family or friend are more likely to exhibit pet food anxiety.*

**Hypothesis 2** **(H2).** *Pet owners who perceive their pet as an animal are more likely to exhibit pet food anxiety.*

**Hypothesis 3** **(H3).** *Pet owners who are actively engaged with their pet are more likely to exhibit pet food anxiety.*

**Hypothesis 4a** **(H4a).** *Pet owners who are older are more likely to exhibit pet food anxiety.*

**Hypothesis 4b** **(H4b).** *Pet owners with lower income are more likely to exhibit pet food anxiety.*

**Hypothesis 4c** **(H4c).** *Pet owners with less education are more likely to exhibit pet food anxiety.*

**Hypothesis 4d** **(H4d).** *Female pet owners are more likely to exhibit pet food anxiety.*

**Hypothesis 5** **(H5).** *Pet owners who more frequently experience problems providing sufficient food for their pets are more likely to exhibit pet food anxiety.*

**Hypothesis 6** **(H6).** *Pet owners who experience pet food anxiety are more likely to exhibit changed shopping behavior in Covidian times.*

**Hypothesis 7** **(H7).** *Pet owners who experience pet food anxiety are more likely to exhibit changed feeding behavior in Covidian times.*

## 2. Material and Methods

### 2.1. Survey Instrument and Data Collection

The data were collected from a sample of US residents targeted in terms of pet ownership. A questionnaire was developed for an online survey that was administered in July 2021. The survey was administered through the survey software Qualtrics and distributed via Amazon Mechanical Turk, a crowdsourcing platform. Respondents had to be US residents and 18 years old to participate. The data collection resulted in 206 completed responses (156 males and 50 female respondents), which were considered appropriate for this research, given that all respondents indicated that they owned a minimum of one cat or dog. The sample of US citizens is appropriate for exploring key factors impacting feeding and shopping behavior via partial least square structural equation (PLS-SEM) modelling, as a standard method in PLS-SEM to determine the minimum sample size has been employed. The “10-times rule” method following Hair et al. (2011), which builds on the assumption that the sample size should be greater than 10 times the maximum number of inner or outer model links pointing at any latent variable in the model [66]. In the proposed model, the maximum number of links was 10 (COVID-19 pet food anxiety with 7 inner and 3 outer), indicating a minimum sample size of 100.

The questionnaire consisted of various sections with fixed response questions where respondents were asked to indicate their interest in pets, pet care and engagement, their experiences with pet food insecurity and anxiety as well as shopping and feeding in Covidian times.

### 2.2. Construct Measurement

While many of the constructs have been discussed in the literature [9,23,25,59], validated scales to adopt for the current research were only partly available. Thus, measurement items were developed from the relevant concepts proposed in the literature. Items using 7-point Likert scales (1 = strongly disagree to 7 = strongly agree) were developed and grouped into scales for pet perceptions and engagement (14 items) adapted from Surie (2014) (e.g., I regularly groom my pet); COVID-19 pet anxiety (3 items) (e.g., Shortages in pet food products makes me feel threatened); pet food shopping changes (5 items) and pet feeding changes (7 items) (e.g., I let my pets roam the neighborhood to find food) are based on Kirk and Rifkin (2017) and Arluke (2021) [25,59,67].

For COVID-19 pet food problem frequency, 4-point frequency scales (1 = never to 4 = often) were employed (e.g., Since COVID-19, how often have you changed the pet food you purchased because that’s all you could find or afford?) [9]. Prior to the model measurement and structure analyses, the 14 pet perceptions and engagement items were subjected to a factor analysis (principle components extraction with varimax rotation) in SPSS, resulting in three factors, which were named pet perceived as friend/family, pet perceived as an animal and active pet engagement.

### 2.3. Data Analysis

The data analysis consisted of descriptive statistics and PLS-SEM. The PLS-SEM method is a combination of path analysis, principal component and regression analysis, and most appropriate to investigating complex causal dependencies of latent constructs in explorative models and coefficient paths [68]. PLS-SEM is particularly appropriate due to a number of characteristics of the current research and data, namely the relatively small sample size and the exploratory nature of the research in a relatively unexplored field. Additionally, the non-normal distribution of the data and combination of Likert scales, frequency scales and single item demographic data preclude the use of maximum likelihood SEM modelling [68]. However, PLS-SEM is not without limitations, one being the inability to test whether relationships are recursive or non-recursive, resulting in models that cannot accommodate reciprocal or circular paths (such as feedback loops) [69].

This research follows what is often called a soft modelling approach, as it applies predictive modelling in the early stages of theory development [70]. As such, analyses are more data driven and results could be overstated or skewed, especially when examined individually and when compared with the theory confirmation role that covariance-based SEM often plays [68]. However, despite its drawbacks, soft modelling is an invaluable tool to examine emerging and rapidly changing issues. Additionally, while the arrows in the model and the wording of hypotheses and results may suggest causality, this is more than can be confirmed in a single cross-sectional dataset; therefore, causal words like influence, impact, lead and heighten should be interpreted with caution as consistency with empirically, logically or theoretically derived causal directions. 

PLS-SEM follows a two-step approach. The first step was dedicated to the outer model and consisted of checking reliability and validity via measurement model functions. Indicator loadings greater than 0.4 verified indicator reliability [68]. In terms of the internal consistency of the model, the average variance extract (AVE > 0.5), construct reliability (Cronbach’s Alpha > 0.6) and composite reliability (CR > 0.6) were used to test the convergence criterion [66,68].

In order to test the constructs within the model, the Fornell–Larcker criterion and cross-loadings determining discriminant validity need to be evaluated. When testing discriminant validity by checking cross-loading, all items should have a higher correlation with their assigned factor than with other factors. The Fornell–Larcker criterion is fulfilled if the square root of each construct’s AVE is greater than the correlation with other constructs [68]. Following Henseler et al. (2015), the heterotrait–monotrait ratio of correlations criterion (HTMT) with a threshold value of 0.9 was used to confirm discriminant validity [71]. Finally, multicollinearity was checked with the Variance Inflation Factor (VIF), which is recommended to be under 5.

The second step is focused on the inner model and aims to determine the structural fit of the model [68]. To evaluate the model quality, the model fit has been reported and the explanatory power was evaluated. Proponents caution the interpretability of model fit indices in SEM-PLS and recommend that all suggested thresholds be applied tentatively; however, convention suggests that goodness-of-fit (GoF) and normed fit index (NFI) are reported and both GoF and NFI scores vary from 0 to 1, where closer to 1 is considered a better fit [68]. Additionally, the standardized root mean square residual (SRMR) has been reported where a value of less than 0.08 is considered acceptable. The explanatory power of the model was evaluated by the individual and average variance explained (R^2^) of the dependent variables, with values of 0.75, 0.5 and 0.25 considered substantial, moderate and weak [68]. Predictive validity was assessed using the Stone Geisser criterion (Q^2^), which, if larger than zero for an endogenous latent variable, the model has adequate predictive relevance for the construct [68]. Furthermore, Q^2^ scores larger than 0.25 and 0.5 indicate medium and large predictive accuracy, respectively. The software packages SPSS and SmartPLS were used to examine the research model and test the proposed hypotheses.

## 3. Results

Table 1 shows the sample description statistics with the median respondent aged 25–34, with a bachelor degree and an annual pre-tax income between $25k and $50k per year.

The assessment of the outer model included the reliability and the convergent and discriminant validity of the scales used to measure the constructs present in the proposed conceptual framework. Table 2 shows that the item factor loadings were all above the minimum 0.4, indicating suitable items in each scale; Cronbach Alpha and composite reliability scores were above 0.6, confirming reliability; and the AVE scores were above 0.5 for all the scales except for active pet engagement (0.492). However, since active pet engagement included a diversity of engagement facets (grooming, daily interaction and knowledge about pet food, health and wellbeing), an AVE of just less than 0.5 was acceptable.

The discriminant validity was tested using the Fornell–Larker criterion and heterotrait–monotrait ratios. Table 3 shows that the requirements of the Fornell–Larker criterion were satisfied, except that the square root of the AVE for pet perceived as an animal was 0.004 less than its cross-loading with COVID-19 pet feeding change. Furthermore, the HTMT ratios confirmed acceptable discriminant validity, except for higher than recommended HTMT ratio between COVID-19 pet food anxiety and COVID-19 shopping changes (1.000) and COVID-19 pet feeding changes (0.965). While concerning, the discriminant validity was largely supported and for those that were not, were considered to be conceptually distinct; therefore, discriminant validity was deemed satisfactory. Finally, multi-collinearity was not seen as a problem as the highest VIF score was 1.825, well below the recommended maximum of 5.

The structure of the conceptual framework was tested, resulting in a goodness of fit of 0.690, a normed fit index of 0.755 and a standardized root mean square residual of 0.078, all indicative of an adequate overall model fit. The tests for the explanatory and predictive power of the conceptual model resulted in R^2^/Q^2^ values of 0.700/0.440 for COVID-19 pet food anxiety, 0.679/0.559 for COVID-19 pet feeding changes and 0.638/0.397 for COVID-19 pet food shopping changes. These results confirm that the explanatory power of the model is moderate to substantial and the predictive accuracy is considered medium to large. Thus, the structure of the model is confirmed to be fit for hypothesis testing.

**Figure 2:**
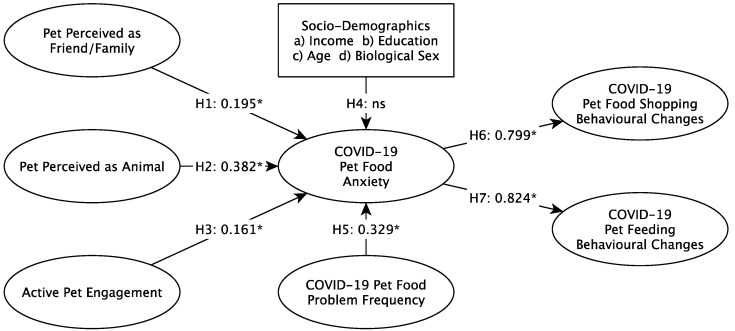
Conceptual Model Results. * *p* < 0.05; ns: not significant.

Table 4 and Figure 2 show the results of the hypothesis testing. Pet perceived as friend/family, perceived as an animal, active pet engagement and frequency of COVID-19 pet food problems all contribute to COVID-19 pet food anxiety, supporting Hypotheses H1, H2, H3 and H5. H4 was not supported, as neither income, education, age nor biological sex were a significant influence on COVID-19 pet food anxiety. H6 and H7 were supported as COVID-19 pet food anxiety influenced COVID-19 changes in pet food shopping and pet feeding behaviors. It should be noted that the (standardized) coefficients for H6 and H7 are very large for marketing research. To this end, it is prudent to consider an alternative to what is a very close alignment of the constructs. Perhaps COVID-19 is such a strong and salient force of change that it is overwhelming those scales, essentially producing a general COVID-19 effect with some attitudinal/behavioral variations.

While biological sex was not found to be a significant predictor of pet food anxiety, there was some concern that the biological sex imbalance in the sample (156 males and 50 females) could have generality or bias implications; therefore, a post-hoc multigroup analysis was performed. A multigroup analysis examines relationship invariance across groups in the sample and the results indicated that the model hypotheses were supported for both sex subgroups, and two of the hypothesized relationships (H6: *p* = 0.040 and H7: *p* = 0.045) were significantly stronger for females.

## 4. Discussion

This study seeks to understand the factors explaining US pet owners feeding and shopping behavior in Covidian times. Overall, the proposed model was found to have an adequate fit and explanatory power. The results emphasize the importance of pet perception, active pet engagement and the frequency that pet owners were unable to provide sufficient food for their pets as factors impacting the owner’s exhibition of pet food anxiety. Sociodemographic factors were not found to have an impact. Pet owners experiencing pet food anxiety are more likely to make changes in feeding and shopping behavior in Covidian times.

The model confirms previous findings that the sociodemographic backgrounds of pet owners are not as relevant as earlier studies on pet ownership and pet food have reported. This finding is consistent with a previous recent study [2]. The study found that attitudes, experience and care for the pet appear to be more important drivers. This can be explained by the fact that in the US, pets are present across all social strata (APPA, 2021), and that the entire nation has had to cope with the disruption of COVID-19 and the resulting adverse effects, such as anxiety and stress, which have led to behavioral changes in shopping and feeding.

It is noteworthy that regardless of whether US pet owners perceive their pet as family or as an animal, they are equally likely to experience pet food anxiety. The classification of animal does not have a negative connotation and does not imply a lack of care and concern for the pet. According to Owns and Grauholz (2019), human–animal relationships can be as complex as human ones. Since the 1950s, the view of what is considered a family in the US has changed multiple times; non-traditional family structures can include interspecies families and thus people who identify themselves as pet parents [72]. Although Owns and Grauholz’s study (2019) revealed that men in particular do not view themselves as pet parents, they use terminology, such as “take care” or “nurture”, and this would imply a strong bond and concern for the pet. People who do not view themselves as pet parents emphasized the duties or chores associated with the pet, and others who were active in the animal rights movement had reservations towards the term pet parenthood for philosophical reasons [72].

Unsurprisingly, pet owners who are actively engaged with their pets are more likely to suffer from pet food anxiety in Covidian times. These owners are likely aware that physical distancing, staying at home and shortages of staple food items, including pet food, somewhat limit optimal engagement. Following Kirk and Rifkin (2020), unpleasant past shopping experiences where pet owners were exposed to panic and competitive buying may have resulted in pet food anxiety [59]. Respectively, the behavioral changes in feeding and shopping can be considered as a strategy to cope with anxiety in challenging times.

## 5. Managerial Implication

The previously discussed findings are of relevance to several participants in the pet industry, particularly animal welfare organizations, veterinaries, managers of animal shelters, food banks and pantries and places where pet owners experiencing pet food anxiety can seek support. Veterinaries and animal welfare organizations could be investing in awareness campaigns and best practice advice related to healthy feeding strategies in the context of food insecurity. This may help to avoid the undesirable feeding practices of skipping days of feeding and suggest which kinds of human food are suitable as a replacement for or to stretch pet food. Furthermore, this may contribute to the prevention of animal neglect.

Managers and volunteers at animal shelters, food banks and pantries may want to consider the findings related to pet food anxiety and shopping. Understanding the psychological and evolutionary reasons behind competitive and panic buying behavior may help to design and facilitate stress and anxiety-free environments and experiences when receiving food assistance. This information is of particular importance to volunteers involved in the operation of these facilities. Volunteers could be trained to better deal with these incidences because, for some, being reliant on these forms of assistance, even under normal circumstances, may be seen as shameful, stressful and have associated stigma. Depending on the extent of need and the availability of resources and volunteers, food and pet food deliveries may be a possibility in periods of high community transmission.

## 6. Future Research and Limitation

The data of the present study was procured using Amazon-Mechanical Turk (Mturk), a well-developed crowd-sourcing platform that has operated for almost a decade. The platform attracted early criticism for its low pricing, but numerous studies have now utilized Mturk for their data collection and have found it to be equal to other forms of samples. According to Goodman and Paolacci (2017), a sample of Mturk workers tends to be more representative of the US population than college samples or in-person or online convenience samples [73]. However, a sample of Mturk workers is perhaps less representative than national probability samples and opt-panels [73,74,75]. Therefore, the expectation is that the relationships of interest investigated in this paper were not strongly biased by the fact that data originated from a crowd-sourcing platform.

Given the conceptual framework of the model, the small number of studies focused on pet food in a context of food insecurity in Covidian times, and the present study addressing a very recent issue, it was felt that the research in its current form still adds value to the existing body of literature, as well as to participants in the pet industry.

Concerning the findings on pet food anxiety, it needs to be acknowledged that living in Covidian times means being exposed to ever-present uncertainty. The uncertainty related to COVID-19 restrictions, resultant panic buying and uncertain availability of pet food products may contribute to more anxiety. Even in an endemic world, the model may still be of relevance given that the emergence of new variants of COVID-19 can lead to anxiety and coping mechanism, such as panic shopping and hording. Similarly, the model may be partially relevant to other natural disasters that result in food and product shortages and the issue of anxiety is present. However, this is subject to future studies.

The specific pet food anxiety context of this study already implies some degree of uncertainty concerning food availability [25]; therefore, this form of anxiety needs to be seen as a genuine form of care. Hopefully, this anxiety does not diminish the contribution and value of service and companion animals towards human wellbeing and mental health.

Future research could focus on the hardship that COVID-19 is imposing on communities, specifically on people’s mental health and wellbeing. Therefore, studies should address preferences for service and companion animals in private and public settings, building on the work of Schoenfeld Tacher et al. (2017) and Gibson and Oliva (2021) [34,35]. Such investigation could use a best–worst approach to understand the tradeoffs pet owners are making when choosing among different alternatives, following Bir et al. (2017) [2]. This approach allows us to understand the tradeoffs consumers are making when choosing between different approaches.

Similarly, further studies may address pet food attribute preferences. This is a rather unexplored area in the academic literature and would allow pet food producers, processors and marketers to develop and adjust product and pet food formulas and match them with consumer needs. Such studies may present a relevant source of information compared to commissioned research conducted by food retailers. With respect to pet food anxiety, further study of consumer willingness to adapt to pandemic-like scenarios and the constructive responses to such by retailers and food assistance agencies are of further relevance.

Following Owns and Grauholz (2019) and Rauktis et al. (2017), the concepts of pet ownership, pet parenting and pet engagement should be studied in different social milieus and family settings [9,72]. Human–animal interaction and the understanding of pet parenting/ownership is likely to be different in households with preschoolers, schoolchildren, teenagers and adult children [38]. A cross-country comparison is of interest as these concepts are influenced by culture and the effects of COVID-19.

Finally, there are many ways that the model could be developed or expanded. While the hypotheses devoted to demographic characteristics were not supported, perhaps the model should examine lifestyle disruptions, such as changes in health, employment status or family situation resulting from COVID-19. Additionally, COVID-19 may have created unanticipated pathways resulting in direct relationships between the exogenous variables and shopping/feeding behavior that operate independent of anxiety.

## 7. Conclusions

This study is relevant to pet food retailers, vets and managers and volunteers at animal shelters, pet food pantries and food banks into the behavioral changes in feeding and pet food buying resulting from pet food anxiety in Covidian times. Partial least squares structural equation modelling showed that perceiving the pet as an animal or family/friend, as well as active engagement with the pet, heightens a sense of pet food anxiety. Similarly, past experiences where pet owners could not provide sufficient food for their pet im-pacts pet food anxiety, which leads to changes in pet food shopping and pet feeding behavior. Sociodemographic factors (biological sex, age, income and education) were not found to impact anxiety.

## Figures and Tables

**Table 1:** Sample description.

**Age**	**Freq**	**%**	**Median**	**StDev**
Under 21	1	0.5		
21–24	9	4.4		
25–34	130	63.1	✓	0.907
35–44	43	20.9		
45–54	14	6.8		
55–64	7	3.4		
65+	2	1		
Total	206	100		
**Education**	**Freq**	**%**	**Median**	**StDev**
Did not finish high school	1	0.5		
Finished high school	20	9.7		
Attended University	23	11.2		
Bachelor Degree	129	62.6	✓	0.826
Postgraduate Degree	33	16		
Total	206	100		
**Household Annual Income**	**Freq**	**%**	**Median**	**StDev**
$0 to $24,999	39	18.9		
$25,000 to $49,999	82	39.8	✓	1.010
$50,000 to $74,999	58	28.2		
$75,000 to $99,999	20	9.7		
$100,000 or higher	7	3.4		
Total	206	100		

**Table 2:** Scale Loadings, Reliabilities and Convergent Validity.

Scales and Items	Loadings	Mean	Min	Max	StDev	CA	CR	AVE
Pet Perceived as Friend/Family						0.825	0.872	0.533
10.2 My pet is my primary companion	0.702	5.825	1	7	1.373			
10.4 I treat my pet like a child	0.714	5.597	1	7	1.331			
10.6 I cannot imagine a life without my pet	0.690	5.539	1	7	1.563			
10.9 I regularly buy treats for my pet	0.631	5.485	1	7	1.309			
10.10 I buy gifts for my pet on special occasions	0.828	5.607	1	7	1.472			
10.11 I regularly buy accessories for my pet	0.780	5.330	1	7	1.526			
Pet Perceived as Animal						0.846	0.896	0.685
10.3 My pet is a nuisance	0.918	4.092	1	7	2.241			
10.5 I treat my pet like an animal	0.682	4.796	1	7	1.823			
10.7 My pet is not a big focus in my life	0.883	4.150	1	7	2.088			
10.13 I know very little about the nutritional value of pet food	0.808	4.777	1	7	1.757			
Active Pet Engagement						0.699	0.786	0.492
10.8 It is important that I interact with my pet every day	0.477	5.947	2	7	1.036			
10.12 I regularly groom my pet	0.584	5.830	1	7	1.154			
10.14 I am confident in my knowledge about pet food	0.886	5.617	2	7	1.297			
10.15 I know that I am feeding my pet food that is best for its health and wellbeing	0.783	5.728	1	7	1.073			
COVID-19 Pet Food Anxiety						0.721	0.845	0.653
38.4 Shortages in pet food products makes me feel threatened	0.902	4.801	1	7	1.838			
38.5 Shortages in pet food products has led me to competitive and/or panic buying behavior	0.898	4.689	1	7	1.853			
38.6 The unavailability of substitute goods makes me anxious	0.582	5.650	1	7	1.351			
Pet Food Problem Frequency (Since COVID-19, …)						0.759	0.862	0.676
42 how often have you worried that you would run out of pet food before being able to buy or receive get more?	0.807	2.306	1	4	0.802			
43 how often have your pets gone without pet food for a day because you couldn’t afford to buy more or were not able to get to the store?	0.866	2.689	1	4	1.017			
45 how often have you changed the pet food you purchased because that’s all you could find or afford?	0.791	2.578	1	4	0.940			
Pet Food Shopping Changes (Since COVID-19, …)						0.859	0.899	0.641
38.1 my pet shopping behavior has changed to include more basic pet food products	0.875	4.811	1	7	1.672			
38.2 my shopping behavior has changed to include more premium pet food products	0.864	4.947	1	7	1.878			
38.3 my shopping behavior has changed to include more bulk pet food	0.778	5.039	1	7	1.722			
38.8 I do more of my pet food shopping online	0.722	5.320	1	7	1.682			
44.1 I stock up on pet food when it is on sale, even though I don’t currently need it	0.754	4.869	1	7	1.640			
Pet Feeding Changes (Since COVID-19, …)						0.966	0.972	0.831
44.2 I feed my pet human food because there is not enough pet food available in my household	0.884	4.646	1	7	2.107			
44.3 I buy less food for myself so that I that I have money to buy pet food	0.899	4.325	1	7	2.067			
44.4 I let my pets roam the neighborhood to find food	0.936	4.248	1	7	2.275			
44.5 I do not pay for other expenses (e.g., car repair, medical expenses, house repairs) so I have money to spend on pet food	0.883	4.165	1	7	2.059			
44.6 I borrow pet food from a friend, neighbor, or relative	0.930	4.277	1	7	2.257			
44.7 I use the animal shelter’s food pantry	0.918	4.374	1	7	2.177			
44.8 I have taken a loan or borrowed money to buy pet food	0.931	4.175	1	7	2.308			

**Table 3:** Discriminant Validity Test Results.

Fornell–Larcker Criterion	Active Pet Engagement	COVID-19 Pet Food Anxiety	COVID-19 Pet Feeding Changes	COVID-19 Pet Food Problem Frequency	COVID-19 Shopping Changes	Pet Perceived as Animal	Pet Perceived as Friend/Family
Active Pet Engagement	0.701						
COVID-19 Pet Food Anxiety	0.507	0.808					
COVID-19 Pet Feeding Changes	0.398	0.824	0.912				
COVID-19 Pet Food Problem Frequency	0.277	0.689	0.731	0.822			
COVID-19 Shopping Changes	0.481	0.799	0.792	0.705	0.801		
Pet Perceived as Animal	0.323	0.716	0.832	0.630	0.700	0.828	
Pet Perceived as Friend/Family	0.625	0.563	0.518	0.378	0.511	0.356	0.730
Heterotrait–Monotrait Ratio							
COVID-19 Pet Food Anxiety	0.618						
COVID-19 Pet Feeding Changes	0.385	0.965					
COVID-19 Pet Food Problem Frequency	0.360	0.885	0.856				
COVID-19 Shopping Changes	0.487	1.000	0.865	0.865			
Pet Perceived as Animal	0.370	0.874	0.898	0.768	0.802		
Pet Perceived as Friend/Family	0.827	0.717	0.552	0.452	0.589	0.399	

**Table 4:** Path Coefficients/Hypothesis Testing Results.

Hypothesized Relationship	Coefficient	T Stat	*p* Value
H1: Pet Perceived as Friend/Family -> COVID-19 Pet Food Anxiety	**0.195**	3.447	0.001
H2: Pet Perceived as Animal -> COVID-19 Pet Food Anxiety	**0.382**	6.304	0.000
H3: Active Pet Engagement -> COVID-19 Pet Food Anxiety	**0.161**	2.890	0.004
H4a: Income -> COVID-19 Pet Food Anxiety	0.024	0.625	0.532
H4b: Education -> COVID-19 Pet Food Anxiety	0.020	0.516	0.606
H4c: Age -> COVID-19 Pet Food Anxiety	−0.018	0.444	0.657
H4d: Biological Sex -> COVID-19 Pet Food Anxiety	0.047	1.327	0.184
H5: COVID-19 Pet Food Problem Frequency -> COVID-19 Pet Food Anxiety	**0.329**	5.280	0.000
H6: COVID-19 Pet Food Anxiety -> COVID-19 Pet Food Shopping Changes	**0.799**	27.260	0.000
H7: COVID-19 Pet Food Anxiety -> COVID-19 Pet Feeding Changes	**0.824**	33.060	0.000

Bold = *p* < 0.01.

## Data Availability

The data presented in this study are available on request from the corresponding author.
